# A novel approach of kinship determination based on the physical length of genetically shared regions of chromosomes

**DOI:** 10.1007/s13258-023-01485-4

**Published:** 2024-01-05

**Authors:** Sohee Cho, Eunsoon Shin, Yoon Gi Park, Seung Ho Choi, Eun Kyung Choe, Jung Ho Bae, Jong-Eun Lee, Soong Deok Lee

**Affiliations:** 1 Institute of Forensic and Anthropological Science, Seoul National University Medical Research Center, Seoul, South Korea; 2 DNA Link, Inc, Seoul, South Korea; 3 Department of Internal Medicine, Seoul National University Hospital Healthcare System Gangnam Center, Seoul, South Korea; 4 Department of Forensic Medicine, Seoul National University College of Medicine, Seoul, South Korea

**Keywords:** Kinship determination, High-density SNP, Korean biobank array, Koreans

## Abstract

**Background:**

Determination of genetic relatedness between individuals plays a crucial role in resolving numerous civil cases involving familial relationships and in forensic investigation concerning missing persons. Short tandem repeats (STRs), known for their high degree of DNA polymorphism, have traditionally been the primary choice of DNA markers in genetic testing, but their application for kinships testing is limited to cases involving close kinship. SNPs have emerged as promising supplementary markers for kinship determination. Nevertheless, the challenging remains in discriminating between third-degree or more distant relatives, such as first cousins, using SNPs.

**Objective:**

To investigate a kinship analysis method for distant degree of familial relationships using high-density SNP data.

**Methods:**

A high-density SNP data from 337 individuals of Korean families using Affymetrix Axiom KORV1.0-96 Array was obtained for this study. SNPs were aligned by chromosomal positions, and identity-by-state (IBS) was determined, and then shared regions as consecutive SNPs with IBS of 1 or 2 were investigated. The physical lengths of these IBS segments were measured and summed them to create an Index, as a measure of kinship.

**Results:**

The kinship was determined by the physical length of shared chromosomal regions that are distinguished by each kinship. Using this method, the relationship was able be distinguished up to the fourth degree of kinship, and non-relatives were clearly distinguished from true relatives. We also found a potential for this approach to be used universally, regardless of microarray platforms for SNP genotyping and populations.

**Conclusion:**

This method has a potential to determine the different degree of kinship between individuals and to distinguish non-relatives from true relatives, which can be of great help for practical applications in kinship determination.

**Supplementary Information:**

The online version contains supplementary material available at 10.1007/s13258-023-01485-4.

## Introduction

Determination of relatedness between individuals is important in resolving practical cases, such as paternity testing, civil disputes regarding familial relationship, and victim identification of mass disasters (Bertoglio et al. 2020). Assessing genetic relatedness through DNA testing is a reliable method of evaluating kinship, which can be performed through pairwise comparisons of individuals. However, in modern society, there is a trend towards nuclear families, which limits the number of family members available to participate in DNA testing for kinship analysis. As a result, there is an increasing need for the involvement of distant relatives in resolving the cases. For example, in Korea, there is a unique situation that distant relatives are required to engage in kinship determination to facilitate family reunions. Since the Korean war in 1950, families have been separated, with some residing in the South, and others in the North. For family reunions, the identification of genetic relationship between individuals from both regions is essential. However, due to the significant passage of time since the division of Korea, there are currently few living individuals who are genetically close relationships, such as parent − child or full-siblings; consequently, it becomes necessary for distant relatives in their next generation to participate in DNA testing.

Short tandem repeats (STRs) have traditionally been chosen as the primary genetic marker in forensic DNA typing for individual identification and paternity testing (Balding and Nichols 1995). These markers can provide sufficient discriminative power for close familial relationships such as paternity or siblings, and increasing the number of markers used can further enhance the reliability of the testing (von Wurmb-Schwark et al. 2015). However, this STR system is less effective for extended familial relationships such as first-cousins, and the inclusion of more loci in the analysis to increase discriminative power is limited by frequent mutational events of STRs and several loci linkages on the same chromosome (von Wurmb-Schwark et al. 2015; Tamura et al. 2015). Additional genetic markers, such as Y chromosomal STRs and mitochondrial DNA polymorphisms can also serve as supplementary tools, but their applicability is limited in cases involving identification of paternal or maternal lineage.

It is no longer surprising that single nucleotide polymorphisms (SNPs) are being used in kinship testing along with STRs (Lareu et al. 2012; Grandell et al. 2016; Mo et al. 2016; Li et al. 2019). They are widely distributed and represent the most prevalent genetic variants in the human genome. The low polymorphism of SNPs that is commonly biallelic can be overcome by high-throughput analysis using microarrays or massively parallel sequencing (MPS) systems (LaFramboise 2009; Davey et al. 2011), allowing the use of a larger number of SNPs in kinship testing analysis (Skare et al. 2009; Kling et al. 2012; Mo et al. 2018). Over the years, commercially available or in-house SNP panels developed for human identification have been evaluated for their usefulness in kinship testing. A set of 94 SNPs from a commercial forensic panel showed an advantage when both STRs and SNPs were combined in calculating the likelihood ratio for kinship (Li et al. 2019). Another panel consisting of 472 SNPs analyzed through MPS could elevate the discriminatory power for second-degree relatives with the specificity and sensitivity of 99.9% and 100%, respectively, compared with 53.7% and 99.9% of 19 commonly used forensic STRs (Mo et al. 2018). Furthermore, the utility of genome-wide SNP genotyping in kinship analysis of second cousins has been demonstrated, which involves counting the number of shared SNPs between two individuals and translating this information into estimated possibilities (Lareu et al. 2012). However, linkage disequilibrium (LD) or linkage of SNPs was not taken into account in this study.

Genetic relatedness can be measured through various approaches that infer identical-by-descent (IBD) between pairs of individuals (Lee et al. 2016; Kling et al. 2019). The Likelihood approach, well-known in forensic genetics, calculates the likelihoods of conditional probabilities based on observing genetic marker data, given a hypothesis about their relatedness. In this approach, population estimates of allele frequencies are required and LD should be taken into account (Weir et al. 2006), which can be challenging in analyses using high-density SNPs. In another approach, the genotypes of individuals are compared, and the segments (shared segments) where both or at least one allele is shared between pairwise individuals are investigated along the chromosomes. The information regarding the investigated shared segments, including the number, position, or length of shared segments, is utilized to distinguish between various relationships (Hill et al. 2013), since these distributions reflect the genetic relatedness of pairs of individuals. For example, the lengths of each shared segment are commonly measured in centimorgan (cM), and the total length of shared segment regions provides a measure of the relationship. A previous study has proposed a method for pairwise kinship analysis based on this approach using a simulated dataset and suggested an index for determining the degree of kinship based on the sum of the genetic length (cM) of shared chromosomal regions (Morimoto et al. 2016). This method has shown the potential to distinguish not only distant relationships up to the fifth-degree of kinship, but also relationships with the same degree of kinship (e.g., uncle–nephew and grandfather–grandson) (Morimoto et al. 2018). Allele frequencies of SNPs were not used and linkage disequilibrium did not need to be considered in this method, but a genetic map to estimate the genetic distance between SNPs within the shared regions were required.

In this study, we investigated a more intuitive approach for pairwise kinship analysis based on the physical length (i.e., megabase; Mb) of the shared segments with allele sharing between individuals, without relying on a genetic map. While the centimorgan, a unit of genetic distance in genetic mapping and linkage analysis, is commonly used to estimate the size of IBD segments in relationship testing, the physical length of shared segments can also provide insights into the degree of kinship, as centimorgans and base pairs largely correspond in humans (Lodish et al. 2004). By using this method, it is possible to exclude potential biases or errors caused by the misspecification of genetic map, even in populations with poorly constructed genetic maps (Daw et al. 2000; Hackett and Broadfoot 2003; Nievergelt et al. 2004). Hence, our aim was to investigate a novel method based on the physical length of shared segments on chromosomes for forensic practical use, utilizing a reference from real family samples rather than simulated data. We evaluated this method using an independent dataset of Korean families and compared it to the previously proposed approach based on genetic distance (Morimoto et al. 2016). Additionally, we discussed potential factors to consider for the practical application of our method in forensic genetics.

## Materials and methods

### Sample description

We collected samples using buccal swabs from 337 individuals of Korean families with claimed pedigree information. Each pair of different familial relationship was categorized with the degree of genetic distance of kinship, and collateral (C) and lineal relationship (L) were separately evaluated considering inheritance patterns. A total of thirteen types of kinship pairs were defined from 47 families consisting of 337 individuals (Table 1). Pairs of non-relatives were randomly generated between unrelated individuals from different families.

**Table 1:** Sample description

Genetic sharing	Category	Relationship	No. of pairs
1st degree (≈ 50%)	L − 1	Parent − Child (P − C)	298
	C − 1	Full siblings (FS)	130
2nd degree (≈ 25%)	L − 2	Grandparent − grandchild (GP − GC)	145
	C − 2	Uncle/aunt − nephew/niece (U − N)	213
3rd degree (≈ 12.5%)	L − 3	Great-grandparent − great-grandchild (GGP − GGC)	10
	C − 3	First cousins (FC)	167
	C − 3	Granduncle/aunt − grandnephew/niece (GU − GN)	57
4th degree (≈ 6.25%)	C − 4	First cousin once removed (FCOR)	84
	C − 4	Great-granduncle/aunt − great-grandnephew/niece (GGU − GGN)	3
5th degree (≈ 3.125%)	C − 5	Second cousins (SC)	40
	C − 5	First cousin twice removed (FCTR)	9
6th degree (≈ 1.563%)	C − 6	Second cousin once removed (SCOR)	17
7th degree (≈ 0.781%)	C − 7	Third cousins (TC)	12
	UN	Unrelated	55,431

For a validation study, we used an independent SNP dataset of 739 Koreans generated using Affymetrix™ Axiom KORV1.0-96 Array comprising > 833 K SNPs (Affymetrix, Santa Clara, CA, USA) (Moon et al. 2019). The data was generated using blood DNA samples. This dataset included 281 pairs of parent–child (P − C), 138 pairs of full siblings (FS), 9 pairs of uncle/aunt–nephew/niece (U − N) relationship, and 1,000 pairs of non-relatives that were randomly generated.

### SNP typing and quality control

Buccal DNA was extracted from collected samples using QIAamp® DNA Investigator kit (QIAGEN, Hilden, Germany), and quantified using Qubit™ 1X dsDNA HS Assay kit with Qubit™ Flex Fluorometer, according to the manufacturer’s instructions (Thermo Fisher Scientific, Waltham, MA, USA). The genotyping was performed using Affymetrix™ Axiom KORV1.1-96 Array comprising > 827 K SNPs (Affymetrix), according to the manufacturer’s instructions (Thermo Fisher Scientific). Raw SNP data was filtered for quality control (QC) using PLINK v1.9 and SNPolisher (Purcell et al. 2007), with the criteria of SNPs with call rate > 0.95, Hardy − Weinberg equilibrium p-value > 1 × 10^− 4^, and minor allele frequency (MAF) cutoffs > 0.1. SNPs on sex chromosomes and duplicated SNPs were excluded. A total of 259,293 SNPs was selected for the subsequent analysis, and haplotypes of these SNPs were estimated using the ShapeIT algorithm (Delaneau et al. 2008). All experiments were performed in accordance with the relevant guidelines and regulations.

### Measurement of the length of shared chromosomal regions

**Fig. 1:**
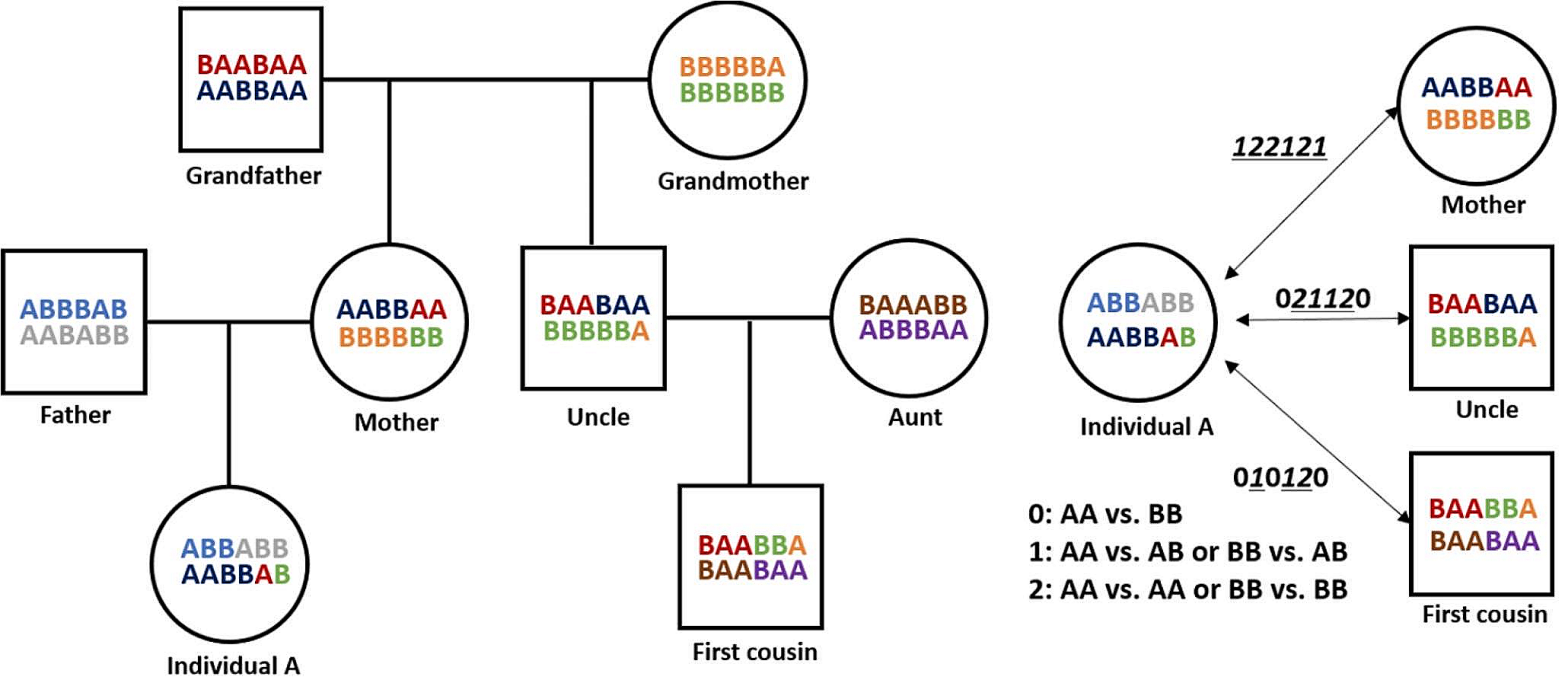
Overview of the method for identifying IBS segments using SNP alleles between two individuals. The shared IBS segments are underlined

We investigated the shared chromosomal regions between two individuals using high-density SNP data. First, the SNPs were aligned according to their chromosomal positions, and then compared for SNP alleles from two individuals to investigate the identity-by-state (IBS). The IBS was determined as 0 when no alleles were shared between two individuals (i.e., AA vs. BB), and 1 or 2 when one or two alleles were shared, respectively (i.e., AA vs. AB or AA vs. AA, respectively). The shared region was then determined by the IBS in which SNPs with IBS of 1 or 2 were consecutive (IBS segment). Uncalled SNPs in either individual were not included in the following analysis. Then, the physical length of the IBS segments was measured by subtracting the chromosomal position of the first SNP locus shared from those of the last SNP locus within the shared IBS segment. The sum of the lengths of shared segments between individuals was used as an index referring kinship, and this index was defined as PD − ICS (physical distance-based index of chromosomal sharing) in this study. Figure 1 provides an overview of this method.

Some of the shared segments investigated by IBS can be coincidental matches, since even unrelated persons could share alleles by chance. To minimize the inclusion of these matches in the calculation, we investigated an optimal threshold (Th) for excluding short segments due to the coincidental matches. In contrast, if this threshold value is too high, true allele shared segments between true relatives can be excluded. The values were investigated by changing the threshold from 0 to 30 Mb, and the performance for kinship discrimination was then evaluated using the area-under-the-curve (AUC) for the receiver operating characteristic (ROC) curve.

The length of the shared segment was also measured based on genetic length between SNPs, as proposed by Ref. (Morimoto et al. 2016). The genetic length was determined as a genetic distance using data from the 1000 Genomes Project (Phase 3), which is the difference between the genetic map position (cM) of the first and last SNP of each shared segment. The sum of the genetic lengths of shared segments was also used an index for kinship determination and was defined as GD − ICS (genetic distance-based index of chromosomal sharing) in this study. The optimal threshold for calculation was also investigated for GD − ICS by changing the threshold from 0 to 30 cM.

## Results

### Physical distance-based ICS distribution for different types of kinship

**Fig. 2:**
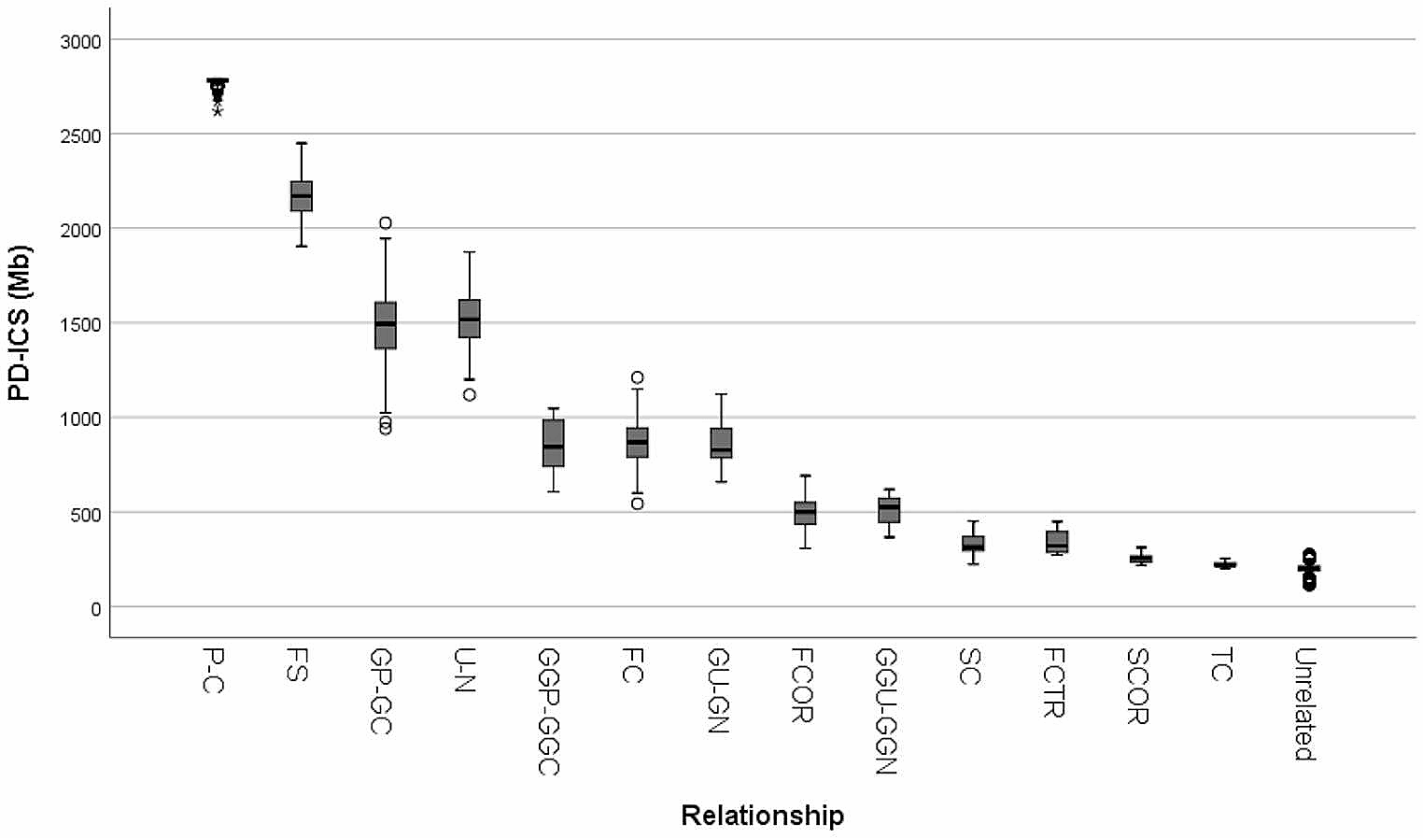
Distributions of PD − ICS values for the investigated Korean families (Th = 3 Mb)

First, we investigated the optimal Th for ICS calculation by assessing the performance in distinguishing various kinships. Through the analysis using the AUC, we determined 3 Mb as the optimal Th, which yielded the highest accuracy of 98.632% (Table S1). The physical lengths of shared segments that exceeded this given threshold were then aggregated to calculate the ICS values based on physical distance. The values for each kinship exhibited distinct distributions, with higher PD − ICS values for closer degrees of genetic relatedness (Fig. 2; Table 2). In particular, unrelated pairs were effectively distinguished from relationships ranging from the first- to the fourth-degree of kinship based on calculated PD − ICS values. However, distant relationships greater than the fifth-degree of kinship had a relatively less portion of being clearly distinguished from the unrelated based on the PD − ICS value. For relationships with the same degree of kinship, such as GP − GC and U − N (second-degree of kinship), or GGP − GGC, FC, and GU − GN (third-degree of kinship), the mean PD − ICS values were very similar, and those relationships with the same degree of genetic sharing were not be distinguished from each other based on the calculated PD − ICS values.

**Table 2:** Statistics of calculated PD − ICS values for the investigated Korean families (Th = 3 Mb).

	P − C	FS	GP − PC	U − N	GGP − GGC	FC	GU − GN	FCOR	GGU − GGN	SC	FCTR	SCOR	TC	Unrelated
No. of pairs	298	130	145	213	10	167	57	84	3	40	9	17	12	55,431
Min	2,617.56	1,904.83	940.35	1,118.60	606.61	543.21	658.14	307.59	365.74	222.89	270.73	217.07	200.33	113.02
Max	2,791.67	2,449.73	2,027.95	1,874.38	1,047.20	1,210.57	1,122.83	691.34	618.75	450.55	449.22	311.33	253.31	274.55
Median	2,782.28	2,170.62	1,494.91	1,518.00	844.00	868.00	827.62	499.91	525.32	314.12	320.36	255.09	217.87	200.78
Mean	2,777.37	2,167.01	1,483.01	1,521.27	839.86	868.89	855.82	499.43	503.27	329.03	340.53	254.99	222.56	199.64
S.D.	17.92	119.68	200.16	141.42	154.68	119.84	112.00	84.44	127.94	54.64	62.79	25.38	15.08	20.00

**Table 3:** Statistics of IBS segments using PD-based ICS method for pairs in second- (A) and third- (B) degree relationship (Th = 3 Mb).

	(A) Second-degree relationship			(B) Third-degree relationship		
	GP − PC	U − N		GGP − GGC	GU − GN	FC
No. of pairs	145	213		10	57	167
Min	33	59		41	41	45
Max	99	117		64	71	91
Median	57	76		51	60	63
Mean	59.44	77.60		51.40	59.46	63.79
S.D.	11.66	10.13		7.44	6.37	8.47

To compare with the PD − ICS method, we calculated the ICS values based on genetic distance (GD − ICS) for the same dataset. First, the optimal threshold for GD − ICS was determined to be 6 cM where the highest accuracy was achieved (99.145%). (Table S2). The GD − ICS values exhibited quite similar distributions to those of PD − ICS values (Fig. S1), and difference of the values between each relationship with the different degree of kinship was also observed (Table S3) as presented in the previous study (Morimoto et al. 2016). Not only the genetic distance of shared segments, but also the physical distance very likely to be utilized for this purpose, and the PD − ICS method can be used for kinship analysis.

### Discrimination of the same degree of kinship using the different pattern of genetic sharing

There was little difference in the mean PD − ICS values for relationships with the same degree of genetic relatedness (e.g., L-2 vs. C-2 or L-3 vs. C-3), lineal and collateral relationships, and these relationships were hardly distinguished only by the values. While the PD − ICS values, which represents the total sum of length of shared segments, are quite similar, differences may exist in patterns that reflect the length and number of shared segments between relationships. This is due to the difference in the frequency of meiosis during the inheritance and the chromosomal transmission from a common ancestor (Hill et al. 2013). The recombination events occur more in the collateral relationship than the lineal relationship; thereby, the IBS segments are shorter in length and larger in number for collateral relationship than lineal relationship.

**Fig. 3:**
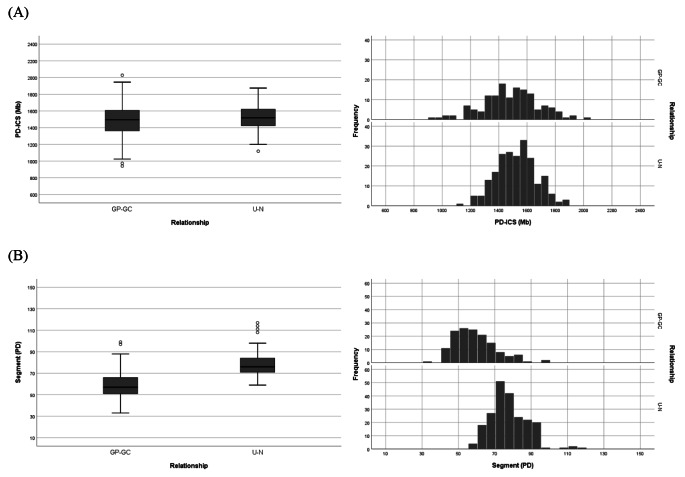
Distributions and frequencies of PD − ICS values (**A**) and of IBS segments (**B**) for pairs in second-degree relationships (GP − GC and U − N)

**Fig. 4:**
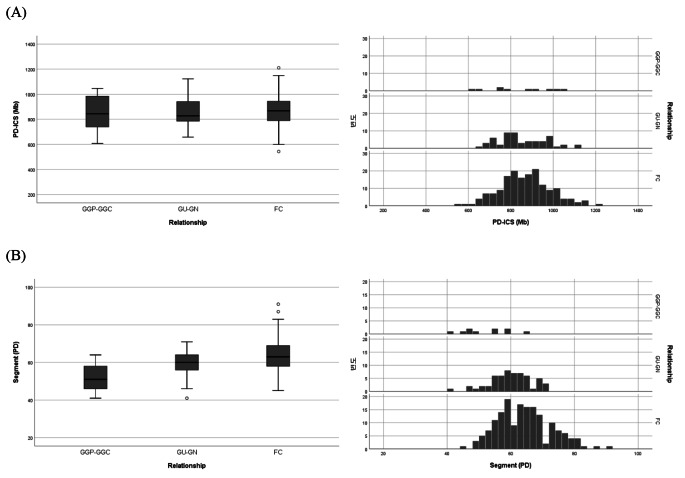
Distributions and frequencies of PD − ICS values (**A**) and of IBS segments (**B**) for pairs in third-degree relationships (GGP − GGC, GU − GN, and FC)

For relationships with second-degree of kinship, GP − GC (L-2) and U − N (C-2), their mean PD − ICS values were not significantly different (Fig. 3A). However, there was a variance in the distribution pattern of the number of IBS segments between PD − ICS value relationships. Specifically, the U − N relationships exhibited a larger number of IBS segments compared to those in GP − GC relationships (Fig. 3B, Table 3A). In the case of relationships with third-degree of kinship, GGP − GGC (L-3), GU − GN (C-3), and FC (C-3), there were no notable differences in the mean PD − ICS values among these relationships (Fig. 4A). However, a slight variation in the number of IBS segments was observed (Fig. 4B, Table 3B). The variation observed in relationships with third-degree of kinships was less pronounced compared to those with the second-degree of kinships, likely attributed to decreased genetic sharing as the degree of genetic relatedness decreased. The PD − ICS method exhibited similar discrimination patterns as the GD − ICS method in this study (Fig. S2 and Fig. S3) and as in previously reported literature (Morimoto et al. 2016).

### Validation of the physical distance-based ICS method with Independent dataset

**Fig. 5:**
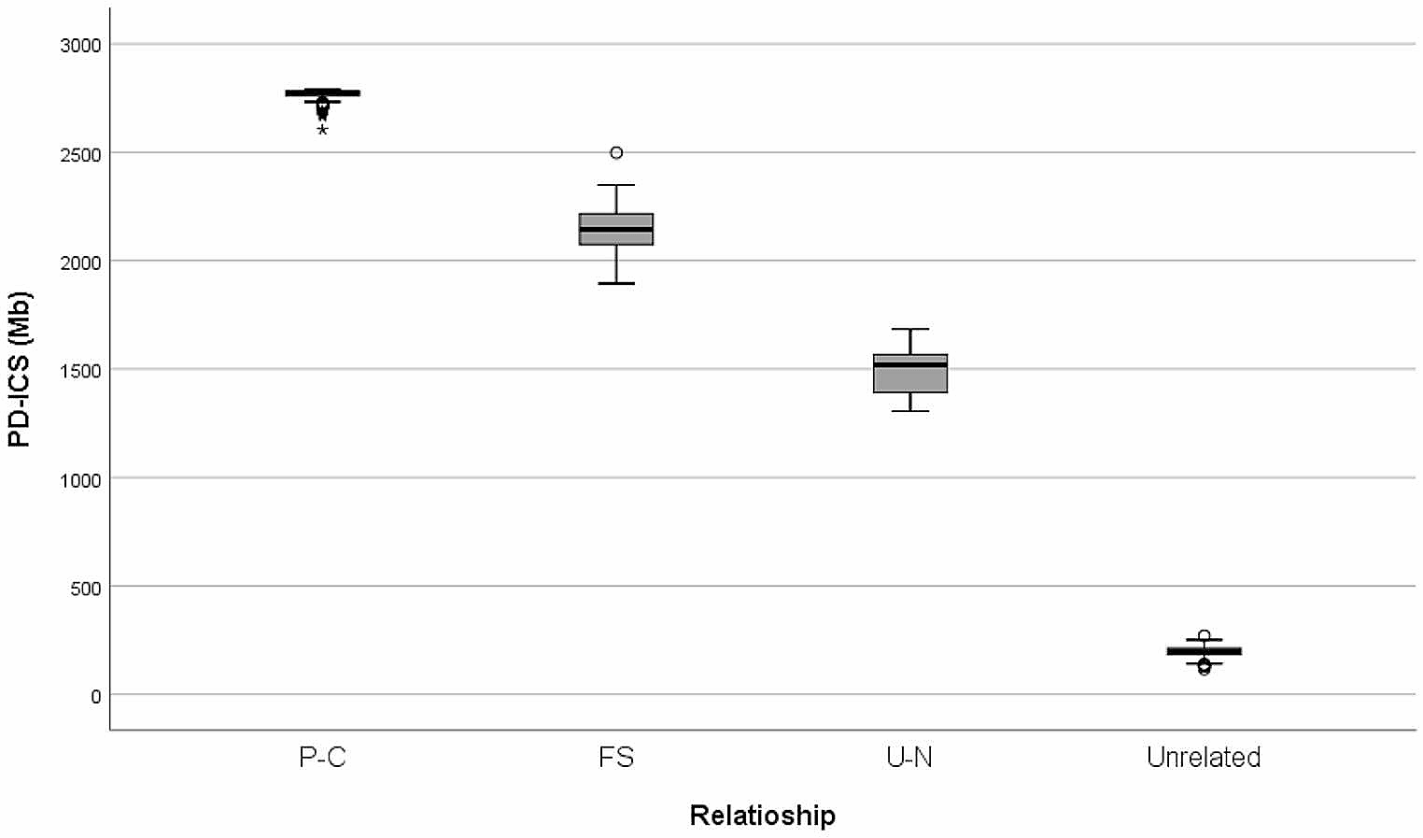
Distributions of PD − ICS values for the validation dataset of Korea families

We conducted a validation of this method using an independent SNP dataset consisting of 833 K SNPs of 739 Korean family members. Relationships of P − C, FS, and U − N all exhibited distinct distributions of PD − ICS values, clearly differentiating them from each other (Fig. 5). Notably, the values were almost identical to those previously observed for P − C (2777.37 ± 17.92 for the investigated dataset vs. 2767.59 ± 23.97 for validation dataset), FS (2167.01 ± 119.68 vs. 2139.06 ± 103.74), and U − N (1521.27 ± 141.42 vs. 1503.15 ± 136.34) (Table 4). This could highlight the reproducibility and reliability of this method.

**Table 4:** Statistics of calculated PD − ICS values for the validation dataset of Korean families (Th = 3 Mb).

	P − C	FS	U − N	Unrelated
No. of pairs	281	138	9	1,000
Min	2,603.96	1,894.65	1,305.65	117.31
Max	2,788.70	2,497.59	1,684.26	270.09
Median	2,775.91	2,143.49	1,518.60	198.20
Mean	2,767.59	2,139.06	1,503.15	197.04
S.D.	23.97	103.74	136.34	21.16

## Discussion

The kinship testing, including paternity testing, has traditionally relied on conventional forensic STR markers. However, its limitations become apparent with those STRs not only in cases involving the determination of relationships with distant degrees (e.g., second-cousins) but also in complex relationships. For example, it is challenging to distinguish full-siblings from half-siblings using autosomal STRs and a standard LR approach (Wen et al. 2022). However, the ICS-based approach offers a valuable means of determining a relationship based on the calculated ICS value without the need for assumptions about the alleged relationship when evaluating genetic evidence. With the same concept, a previous study suggested a promising and practical kinship analysis method that measures the genetic length of shared chromosomal segments between individuals, utilizing high-density SNP data (Morimoto et al. 2016). In this approach, allele or haplotype frequencies of SNPs were not required. Here, we presented a more intuitive and straightforward approach. It measures the physical lengths of IBS segments using chromosomal positions of SNPs instead of genetic distance, without the genetic mapping. The ICS values using this method exhibited distinct distributions that varied with the degree of genetic relatedness for each type of kinship (Fig. 2) and were validated using an independent dataset from Koreans (Fig. 5). It was also confirmed that differences in the number of IBS segments for lineal and collateral relationships (Figs. 3 and 4). These findings suggest that our approach is applicable for analyzing various relationships, including those with distant degrees of kinship. Interestingly, the PD − ICS and GD − ICS values for the investigated dataset seem to support the idea that in humans 1 cM on average corresponds to approximately 7.5 × 10^5^ bp (Lodish et al. 2004). While genetic distance is a well-established measure in studies utilizing GWAS data (Browning and Browning 2013), the genetic distance in specific regions of the genome might be imprecisely estimated due to the high recombination frequency of SNP loci (Robinson 1998; Myers et al. 2005). Additionally, the method is valuable for populations in which genetic maps for estimating genetic distances are less developed or non-existent.

We applied this method to SNP data from samples obtained using two distinct sample collection methods: buccal swabs (buccal DNA) and venipuncture (blood DNA, validation dataset). The quality of extracted DNA may vary depending on the source of DNA or the sample collection method, which can influence the performance of microarray-based genotyping. For instance, DNA degradation or DNA contamination by oral microorganisms might impact the performance of the buccal DNA in microarray-based genotyping compared to the blood DNA, potentially resulting in variations in valid SNP calls (Livy et al. 2012; Samson et al. 2020). Previously, we conducted preliminary tests to evaluate the influence of DNA on this ICS method regarding the quantity of input DNA and the source of DNA. According to the manufacturer’s guidelines of the microarray, it is recommended to use SNP data obtained with a dish quality control (DQC) > 0.82, call rates > 97%, and normal hetero rates (around ~ 15%) for subsequent analysis. In our results, most SNP calls from both samples met those criteria, but blood DNA had a higher number of valid SNP calls compared to buccal DNA. Blood DNA slightly outperformed buccal DNA in genotyping with a DQC of 0.988 ± 0.002 (compared to 0.965 ± 0.014 for buccal DNA), a call rate of 99.3 ± 0.3% (compared to 99.2 ± 0.3% for buccal DNA), and a hetero rate of 14.8 ± 1.1% (compared to 15.2 ± 0.3% for buccal DNA). In the result of ICS distribution, a slightly greater variation in PD − ICS values was observed in the buccal DNA samples compared to those from blood DNA in the validation dataset (Figs. 2 and 5). For DNA quantity, 50 ng of blood DNA showed comparable DQC value and call rates of SNP calls to those achieved with 200 ng of blood DNA and almost identical distribution of PD − ICS values (Fig. S4). Moreover, even as little as 10 ng of blood DNA yielded nearly identical PD − ICS values in comparison to 200 ng of blood DNA. This suggests that the sources of DNA may have influenced the variances in ICS values. Other potential contributing factors, such as the use of different SNP sets or varying quantity of SNPs, will also be further explored in future studies. In the field of practice, DNA samples in a small amount or low quality may be employed for analysis under various circumstances, which is common in forensic casework. As a substantial amount of DNA is generally recommended for microarray-based genotyping on commercial platforms, it is crucial to investigate the optimal DNA quantity and quality for obtaining reliable results in forensic applications.

The outcomes of studies utilizing high-density SNP data can be affected by the traits of the SNPs employed (Wollstein et al. 2007). The SNP array utilized in this study was the Axiom Korean Biobank array aiming to provide enhanced genomic coverage for the Korean population (Moon et al. 2019). This array is comprised of > 833 K SNP markers selected from > 2,500 sequencing data in Koreans, which is expected to be more representative for Koreans. In our preliminary study, we tested this method to SNP data generated using the Affymetrix genome-wide microarray platform (Genome-wide human SNP Array 6.0). This platform is one of the widely used commercial arrays designed for multiethnic populations, and it might have fewer SNPs relevant to Koreans. Despite differences in the platforms used and the SNPs selected following the QC process prior to ICS calculation, there was little variance in PD − ICS values between the two platforms (data not shown). This suggests the potential universal applicability of this approach for high-density SNP data generated from microarrays, regardless of the particular SNPs on the array. However, since the number of SNPs available for analysis after QC can vary, the optimal number of SNPs for ICS calculation should be investigated in further study. This is particularly important in forensic sample analysis, where the SNP call rate can be fluctuated depending on sample quality.

We also compared the PD − ICS method and GD − ICS method for ICS calculation using the same SNP data. The distributions of ICS values by two methods exhibited similar power for distinguishing various relationships (Fig. 2 and Fig. S1). Additionally, we compared the distribution of GD − ICS values in our study with the ICS variability observed in the previous study (Morimoto et al. 2016). In that study, the ICS method was developed using 174,254 SNP loci selected from HumanCore-12 BeadChip or HumanCore-24 BeadChip (Illumina) to construct 249 pedigrees for 1,498 Japanese individuals as founders of haplotypes in simulation. Interestingly, the calculated GD − ICS values exhibited comparable distribution patterns in both studies, even though several factors differed, including the populations (Koreans vs. Japanese), the selected SNP loci and their number for analysis, the source of SNP data (actual DNA samples vs. simulation), and the microarray platform applied for genotyping. This finding could support the versatility of the GD − ICS method. However, further studies still required expanded validation in lager populations, including non-Asians, as these studies were limited to East Asian origins, which may be genetically similar (Kim et al. 2005). Furthermore, using different SNP datasets could result in variations in ICS value, which may be necessary to investigate a more appropriate threshold value. Thresholds have the potential to impact the differentiation of ICS values among relationships with varying degrees of kinship, potentially resulting in variations in ICS values across different datasets (Fig. S5). Additionally, in practical applications, it is necessary to establish a statistical method for evaluating the likelihood or probability of kinship with some measures such as sensitivity, specificity, and error rate, considering the optimal threshold value and the specific set of SNPs employed in the analysis.

The main purpose of this study was to introduce a useful kinship analysis method for forensic applications. We adopted the established correlation between genetic distance and physical distance in this approach and developed the method. This method not only demonstrated the usefulness in distinguishing relationships with different degree of kinship but also in detecting relatives from non-relatives. In practical applications, it is more common to encounter cases where it is needed to distinguish relationships with similar generational differences, such as parent-child or uncle-nephew relationship, and this method is effective in resolving those relationships. We believe that this method will provide a help in cases that are difficult to resolve using the conventional DNA markers in kinship analysis.

### Electronic supplementary material

Below is the link to the electronic supplementary material.

Supplementary Material 1

## Data Availability

The datasets used and/or analyzed during the current study are available from the corresponding author on reasonable request.
