# Supplementary material for: A novel approach of kinship determination based on the physical length of genetically shared regions of chromosomes
Source: Genes Genomics. 2024 Jan 5;46(5):577–87. doi: 10.1007/s13258-023-01485-4 (PMC11024047; doi:10.1007/s13258-023-01485-4)

**Table S1 The mean AUC of threshold for PD-based ICS calculation.**

| Threshold (Mb) | Mean AUCs | Rank |
| --- | --- | --- |
| 0_Th | 0.93922 | 27 |
| 1_Th | 0.97612 | 11 |
| 2_Th | 0.98173 | 5 |
| ***3_Th*** | ***0.98632*** | ***1*** |
| 4_Th | 0.98515 | 2 |
| 5_Th | 0.98327 | 3 |
| 6_Th | 0.98271 | 4 |
| 7_Th | 0.98156 | 6 |
| 8_Th | 0.97687 | 8 |
| 9_Th | 0.97729 | 7 |
| 10_Th | 0.97635 | 10 |
| 11_Th | 0.97652 | 9 |
| 12_Th | 0.97434 | 12 |
| 13_Th | 0.97301 | 13 |
| 14_Th | 0.97077 | 14 |
| 15_Th | 0.96298 | 17 |
| 16_Th | 0.96268 | 19 |
| 17_Th | 0.96460 | 16 |
| 18_Th | 0.96667 | 15 |
| 19_Th | 0.96294 | 18 |
| 20_Th | 0.96011 | 20 |
| 21_Th | 0.95626 | 21 |
| 22_Th | 0.95074 | 22 |
| 23_Th | 0.94684 | 23 |
| 24_Th | 0.94643 | 24 |
| 25_Th | 0.94095 | 26 |
| 26_Th | 0.94370 | 25 |
| 27_Th | 0.93912 | 28 |
| 28_Th | 0.93615 | 29 |
| 29_Th | 0.93411 | 30 |
| 30_Th | 0.93325 | 31 |

**Table S2 The mean AUC of threshold for GD-based ICS calculation.**

| Threshold | Mean AUCs | Rank |
| --- | --- | --- |
| 0_Th | 0.96093 | 25 |
| 1_Th | 0.97807 | 15 |
| 2_Th | 0.98492 | 10 |
| 3_Th | 0.98768 | 6 |
| 4_Th | 0.98754 | 7 |
| 5_Th | 0.99018 | 3 |
| ***6_Th*** | ***0.99145*** | ***1*** |
| 7_Th | 0.98959 | 4 |
| 8_Th | 0.99024 | 2 |
| 9_Th | 0.98938 | 5 |
| 10_Th | 0.98708 | 8 |
| 11_Th | 0.98563 | 9 |
| 12_Th | 0.98431 | 11 |
| 13_Th | 0.98264 | 12 |
| 14_Th | 0.98165 | 13 |
| 15_Th | 0.98087 | 14 |
| 16_Th | 0.97650 | 16 |
| 17_Th | 0.97369 | 17 |
| 18_Th | 0.97282 | 18 |
| 19_Th | 0.97102 | 20 |
| 20_Th | 0.97104 | 19 |
| 21_Th | 0.97092 | 21 |
| 22_Th | 0.97029 | 22 |
| 23_Th | 0.96884 | 23 |
| 24_Th | 0.96745 | 24 |
| 25_Th | 0.95699 | 26 |
| 26_Th | 0.95574 | 27 |
| 27_Th | 0.95404 | 28 |
| 28_Th | 0.95216 | 29 |
| 29_Th | 0.95037 | 30 |
| 30_Th | 0.92246 | 31 |

**Table S3 Statistics of calculated GD−ICS values for the investigated Korean families (Th=6 cM).**

|  | P−C | FS | GP−GC | U−N | GGP−GGC | FC | GU−GN | FCOR | GGU−GGN | SC | FCTR | SCOR | TC | Unrelated |
| --- | --- | --- | --- | --- | --- | --- | --- | --- | --- | --- | --- | --- | --- | --- |
| No. of pairs | 298 | 130 | 145 | 213 | 10 | 167 | 57 | 84 | 3 | 40 | 9 | 17 | 12 | 55,431 |
| Min | 3,272.85 | 2,400.93 | 1,148.66 | 1,268.41 | 640.05 | 531.50 | 651.51 | 209.20 | 294.17 | 67.75 | 123.36 | 54.98 | 47.34 | 0.00 |
| Max | 3,622.09 | 3,071.71 | 2,464.09 | 2,330.13 | 1,171.71 | 1,409.40 | 1,261.09 | 757.58 | 482.55 | 373.88 | 322.57 | 134.67 | 96.47 | 81.77 |
| Median | 3,607.25 | 2,739.48 | 1,825.66 | 1,838.40 | 920.92 | 935.49 | 929.37 | 451.23 | 417.14 | 214.80 | 177.52 | 100.64 | 59.35 | 46.95 |
| Mean | 3,596.51 | 2,746.93 | 1,813.86 | 1,843.82 | 915.16 | 949.56 | 938.65 | 458.62 | 397.96 | 225.06 | 202.79 | 99.50 | 64.12 | 44.18 |
| S.D. | 36.67 | 144.58 | 250.79 | 184.46 | 176.74 | 157.98 | 139.21 | 112.15 | 95.64 | 77.95 | 66.15 | 22.08 | 15.15 | 10.37 |

**Fig. S1 Distribution of GD−ICS values for the investigated Korean families (Th= 6 cM).**


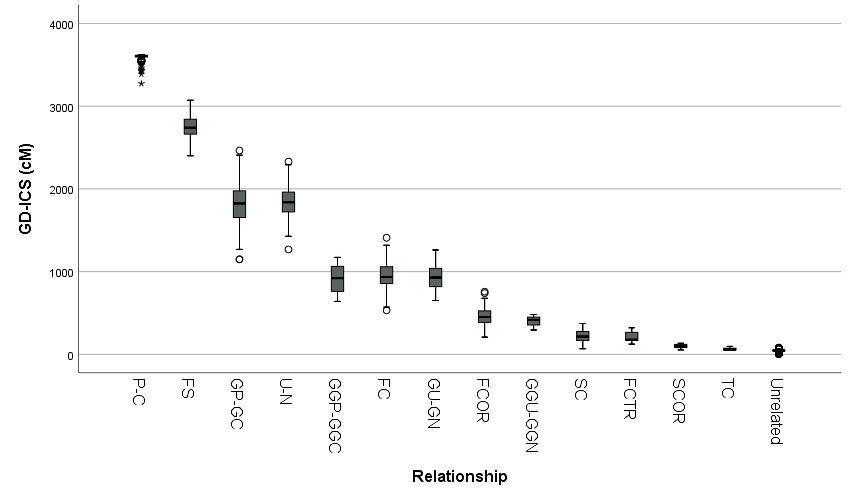


**Fig. S2 Distributions and frequencies of GD−ICS values (A) and of IBS segments (B) for pairs in second-degree relationships (GP−GC and U−N).**

(A)


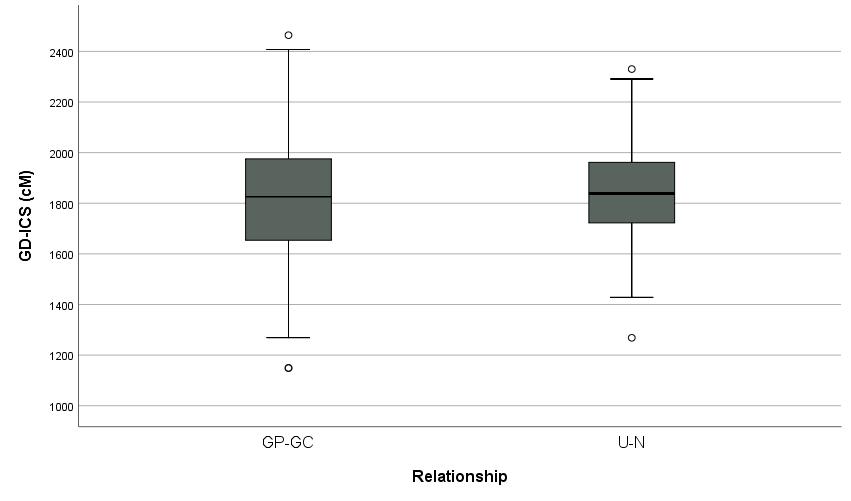

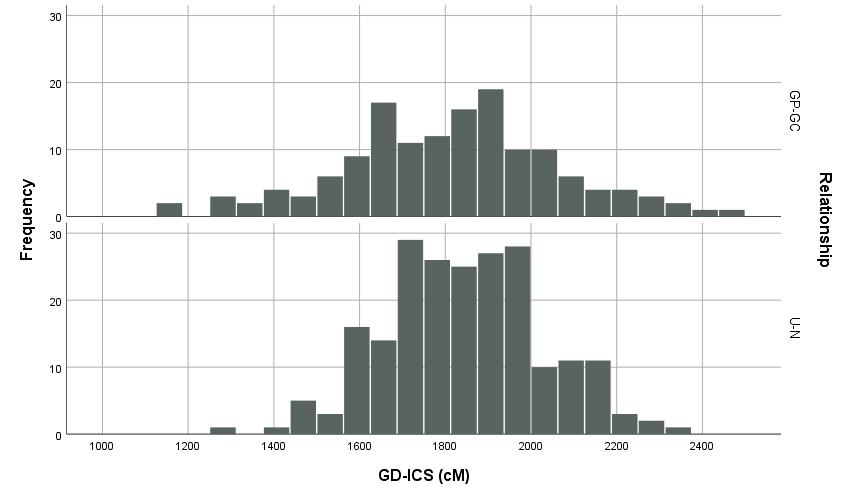


(B)


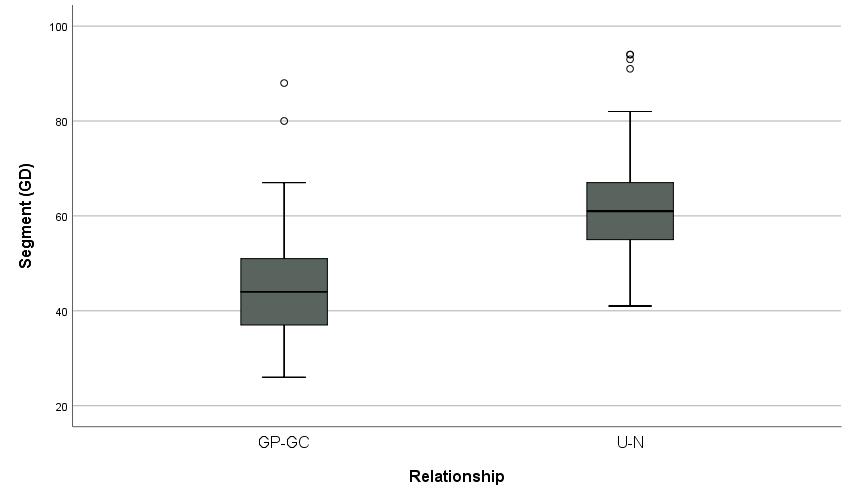
 **
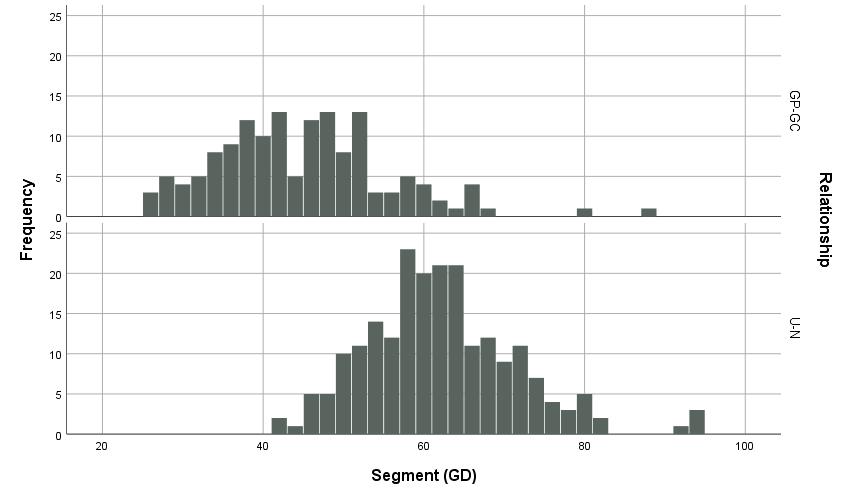
**

**Fig. S3 Distributions and frequencies of GD−ICS values (A) and of IBS segments (B) for pairs in third-degree relationships (GGP−GGC, GU−GN, and FC).**

(A)


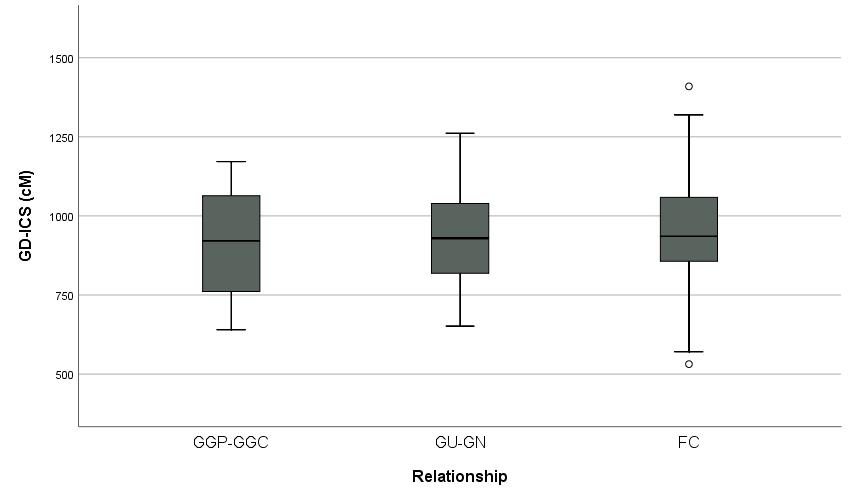

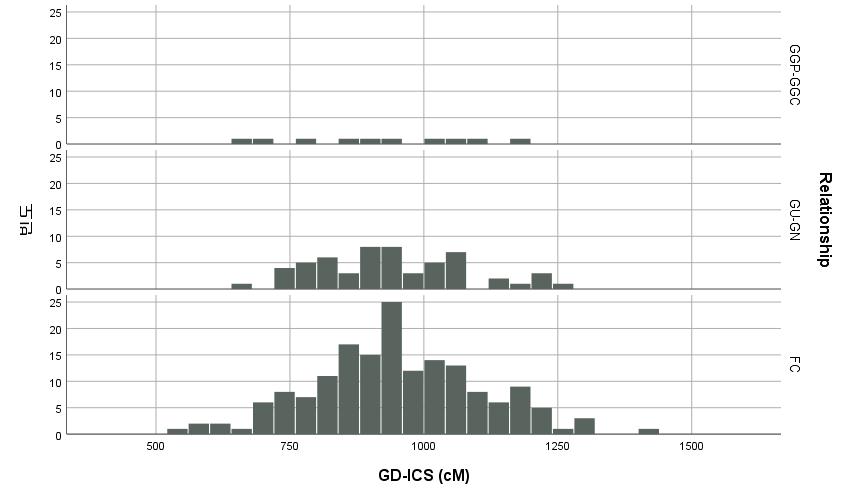


(B)


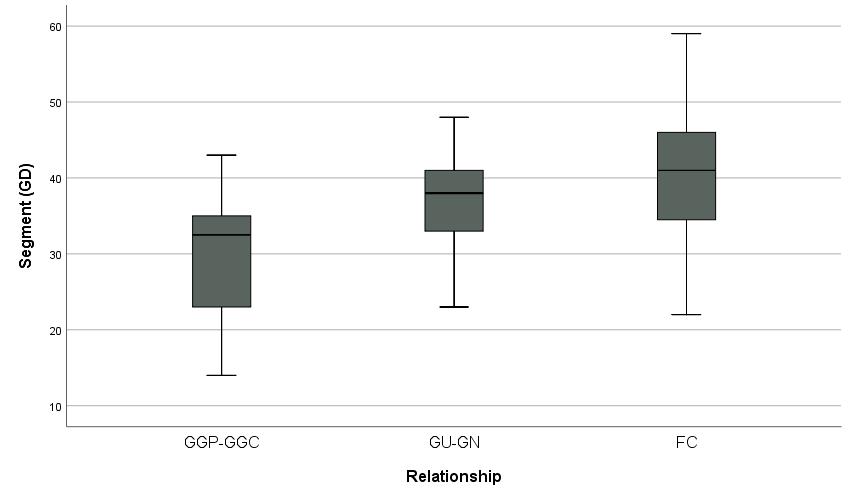

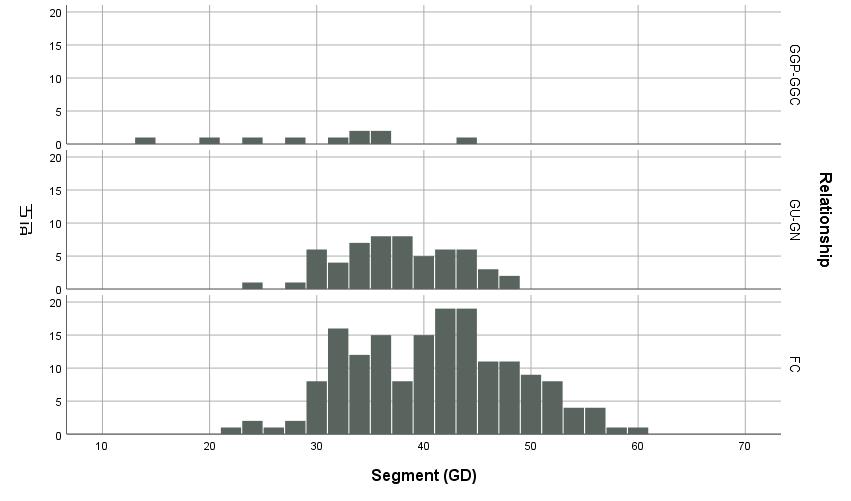


**Fig. S4 Distributions of PD−ICS values for (A) P−C pairs, (B) FS pairs, (C) U−N pairs, and (D) FC pairs of relationships (Th= 3 Mb) with 200 ng, 100 ng, 50 ng, 10 ng, and 2 ng of input DNA in the preliminary study. The values for each relationship were reference (200 ng of DNA) from Fig. 2.**

(A) (B)


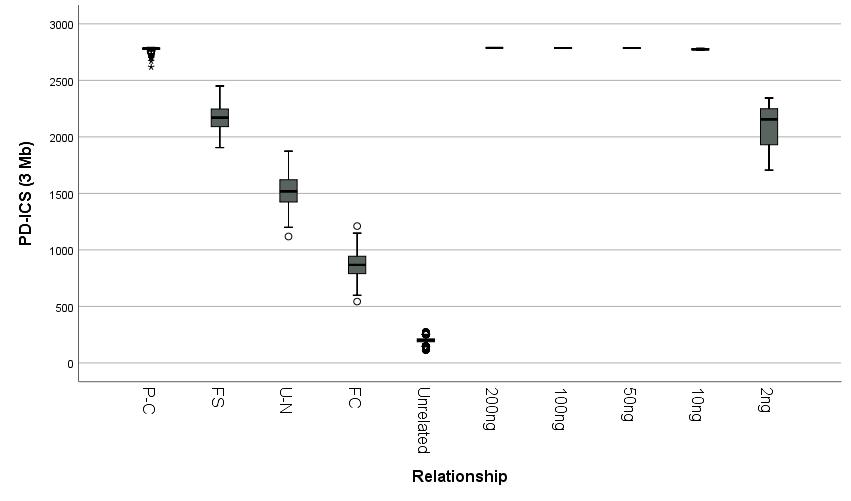

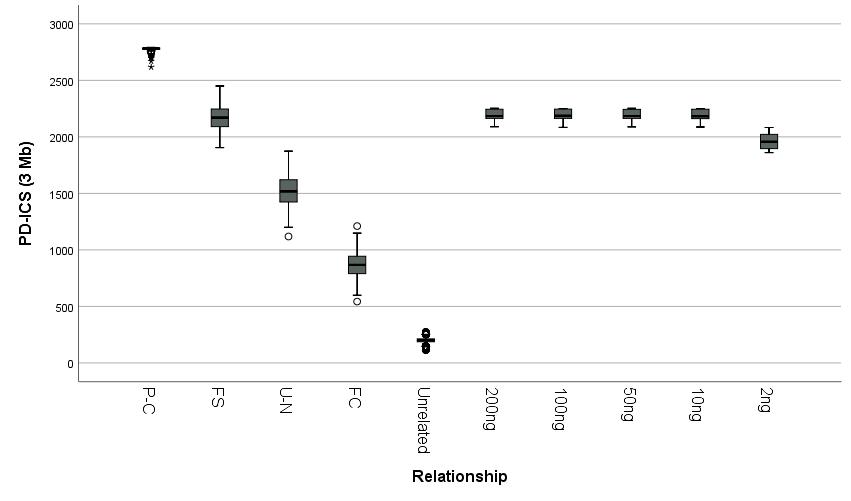


(C) (D)


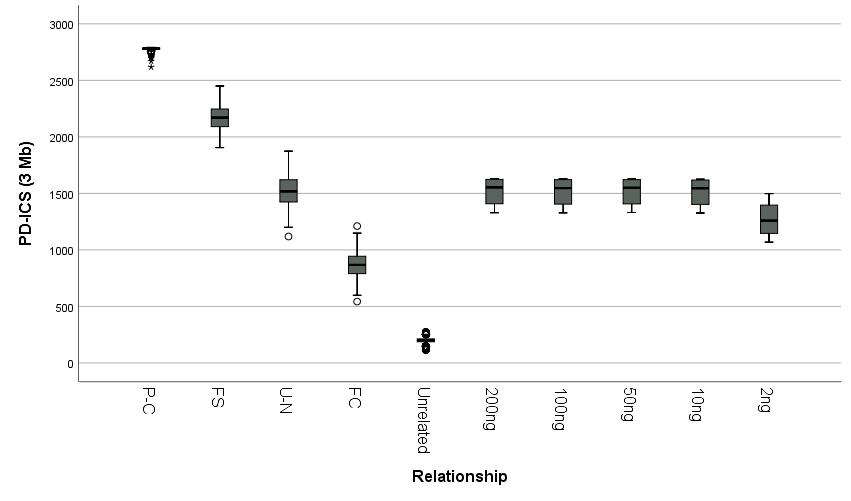

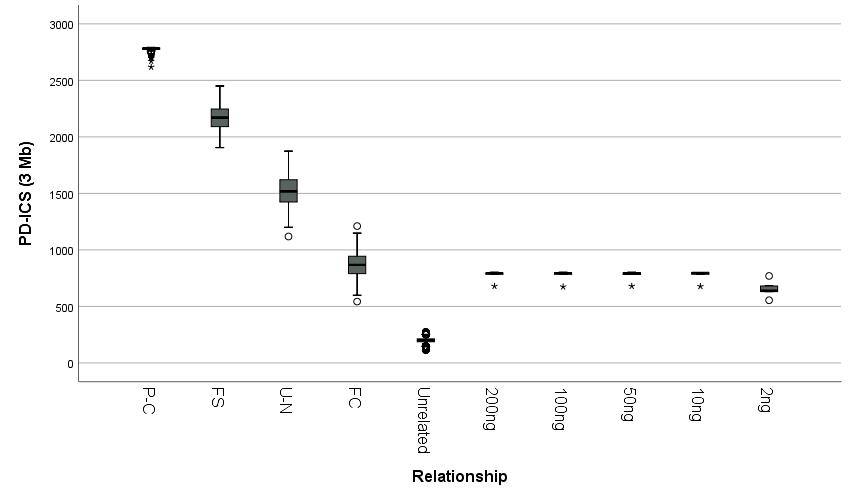


**Fig. S5 Distributions of PD−ICS values for the investigated Korean families with Th= 6 Mb (A), 9 Mb (B), and 12 Mb (C).**

(A)


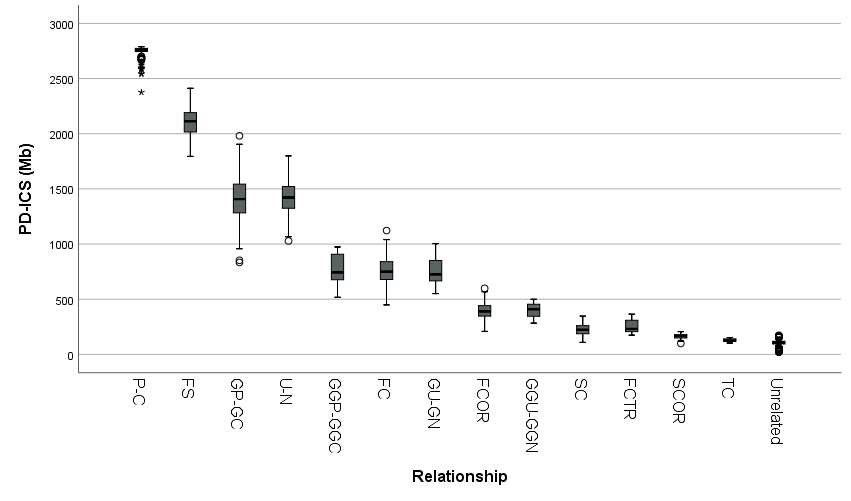


(B)


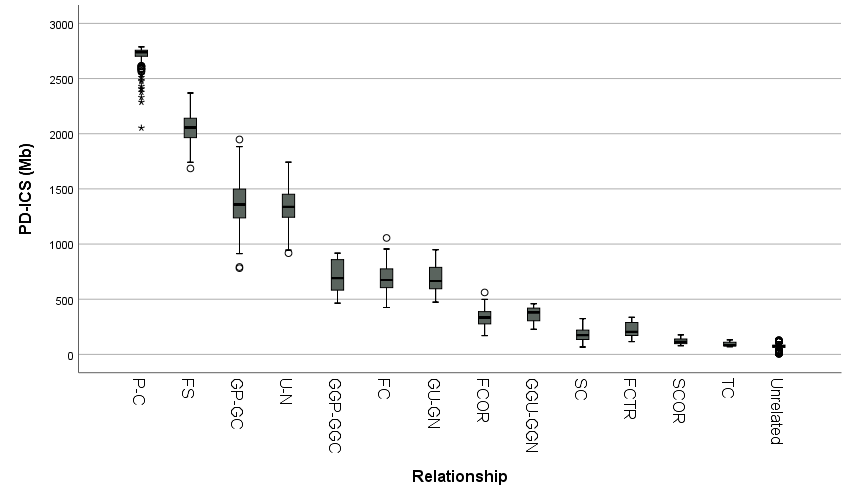


(C)


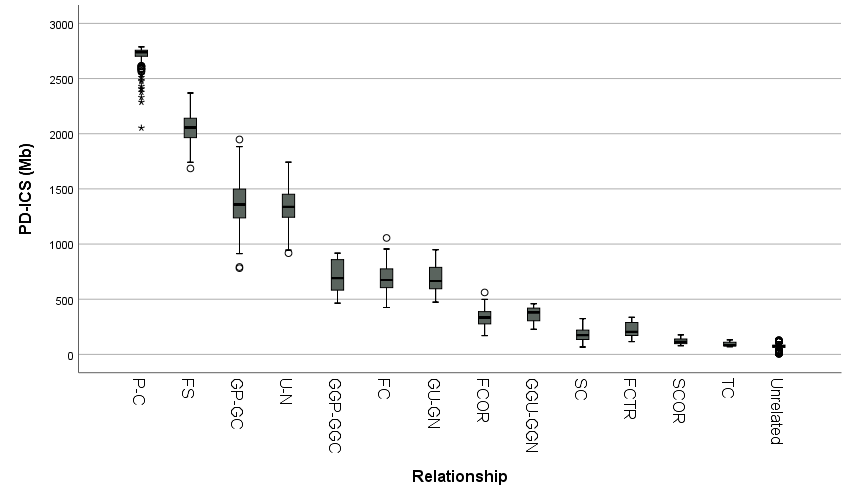

Supplement: Supplementary file 1 — Supplementary Material 1 [file 13258_2023_1485_MOESM1_ESM.docx]
